# *Candida albicans*: A Major Fungal Pathogen of Humans

**DOI:** 10.3390/pathogens11040459

**Published:** 2022-04-11

**Authors:** Jonathan P. Richardson

**Affiliations:** Centre for Host-Microbiome Interactions, Faculty of Dentistry, Oral & Craniofacial Sciences, King’s College London, London SE1 1UL, UK; jonathan.richardson@kcl.ac.uk

Fungal infections kill ~1.6 million people every year [1]. The fungal pathogen *Candida albicans* causes > 150 million mucosal infections and ~200,000 deaths per annum due to invasive and disseminated disease in susceptible populations. Economically, yearly healthcare costs for *Candida* infections in the USA are ~$2 billion [2], with similar per capita costs in the European Union. *C. albicans* accounts for ~75% of all *Candida* infections and is an enormous global health burden, the severity of which continues to escalate.

This Special Issue, “*Candida albicans*: A Major Fungal Pathogen of Humans”, combines review articles [3,4,5,6,7] and original research [8,9,10,11,12] to explore recent advances in our understanding of *C. albicans* morphological switching, nutrient acquisition and metabolism, invasive infection in neonates, antimicrobial host responses and the development of potential therapeutics.

Morphological plasticity is considered a central tenet of *C. albicans* pathogenicity. While numerous forms of *C. albicans* are known to exist including chlamydospores, gray cells, GUT (gastrointestinal induced transition) cells, and white/opaque cells, the reversible yeast-to-hypha transition is widely regarded as a crucial weapon in the *C. albicans* arsenal. The intracellular signaling networks that activate morphological switching and the maintenance of sustained hyphal growth in response to diverse environmental cues are explored by Chow et al. [3]. The importance of chromatin-mediated epigenetic regulation in *C. albicans* morphological switching and biofilm formation is highlighted by Iracane et al. [4], in which an overview of *C. albicans* chromatin structure, histone modification, chromatin remodeling, and the influence of non-coding transcription and non-coding RNAs is presented.

*C. albicans* must assimilate nutrients acquired from a hostile host environment in order to thrive and persist; a process which occurs in the face of an often competitive microbiota. The versatility of amino acids as a nutrient source is addressed by Silao et al. [5], in which nutrient sensing, amino acid uptake and metabolism are explored with a particular emphasis on proline catabolism. Cellular metabolism is an important component of host–microbe interplay during commensal colonization and infection. In their review, Pellon et al. [6] discuss the metabolic flexibility of *C. albicans* in the context of commensalism and virulence, and the role of host metabolism in the control of innate immune responses to fungal infection.

Invasive fungal disease is a major cause of infection-related death among critically ill newborns. The risk factors associated with the development of invasive *Candida* infections following major surgery in neonates are investigated by De Rose et al. [7]. The authors also discuss fungal colonization of preterm infants, innate neonatal defence, and explore the epidemiology of fungal infection in neonatal intensive care units and the diagnosis and financial burden of invasive *Candida* infections together with prophylaxis.

The production of antimicrobial proteins is a key feature of host defence against active fungal infection. Research by Dishman et al. [8] provides an intriguing glimpse into the mechanistic action of “metamorphic proteins”; molecules that can reversibly switch between alternative structural conformations. One such protein is XCL1; a human chemokine capable of killing *Escherichia coli* and *C. albicans*. By locking XCL1 into distinct three-dimensional structures, Dishman et al. demonstrate that different conformations of XCL1 kill *C. albicans* in vitro via two different mechanisms. Such intriguing findings may one day inform on the design of future therapeutics.

The unacceptably high mortality rate associated with invasive fungal infections is a reminder of the current shortcomings in antifungal therapy. A limited number of antifungal drugs combined with ever-increasing levels of antifungal resistance highlights the desperate need for new avenues of therapeutic intervention. In their article, Faria et al. [9] explore the antifungal activity and in vivo toxicity of a 1,3,4-oxadiazole derivative (LMM6). Application of LMM6 to *C. albicans* in vitro revealed encouraging antifungal and anti-biofilm activity, and reduced fungal burdens (kidney, spleen) in a murine model of systemic candidiasis.

The torsional stress experienced by DNA during replication and transcription is relieved by topoisomerase activity. Fungal DNA topoisomerases are highlighted as a potential antifungal target by Gabriel et al. [10], who demonstrate that capridine-β accumulates in *C. albicans* cells where it undergoes subsequent biotransformation into an inhibitor of fungal topoisomerase II activity in a strain-dependent manner.

Sphingolipids have recently garnered attention as potential targets for antifungal therapy due to their central role in fungal growth, morphogenesis, and virulence. Aureobasidin A and myriocin inhibit inositol phosphorylceramide synthase and glucosylceramide synthase, respectively, which are enzymes required for sphingolipid synthesis in fungi. In their article, Rollin-Pinheiro et al. [11] evaluate the antifungal activity of Aureobasidin A and myriocin against type strains and fluconazole-resistant clinical isolates of *C. albicans* and *C. glabrata*. Both compounds displayed encouraging antifungal activity in vitro and functioned synergistically with fluconazole, highlighting potential routes toward combinatorial therapy.

Attachment of *C. albicans* to epithelial cells is a prerequisite for commensal colonization and pathogenic infiltration of mucosal barriers [13]. Chemical strategies to reduce physical interaction between pathogenic fungi and host cells are yielding encouraging results. In their article, Martin et al. [12] describe the synthesis of a multivalent glycoconjugate in which an inhibitor of *C. albicans* adhesion is chemically coupled to a linear peptoid scaffold. Fungal adherence to buccal epithelial cells was reduced in vitro following treatment with the glycoconjugate formulation, and investigations to elucidate the precise mechanism of action are currently ongoing.

Despite significant advances in our understanding of *C. albicans* biology and immunopathology, selective pressures within the host environment continue to mould *C. albicans* into an ever-more formidable foe. Improvements in the breadth of the antifungal armoury are required if we are to overcome the challenges associated with increasing levels of resistance. Continued research aims to pave the way towards more positive patient outcomes.

I thank all of the authors that contributed to this Special Issue of *Pathogens*. Your time and expertise are appreciated.
